# Analysis of Equity Disputes in Listed Companies With Dispersed Ownership Structure and Protection of Small and Medium Shareholders’ Interests

**DOI:** 10.3389/fpsyg.2022.857585

**Published:** 2022-05-20

**Authors:** Chun Xi He, Wei Ni Soh, Tze San Ong, Wei Theng Lau, Bin Zhong

**Affiliations:** School of Business and Economics, University Putra Malaysia, Serdang, Malaysia

**Keywords:** small and medium shareholders, dispersed ownership structure, equity disputes, interest protection, corporate governance

## Abstract

This paper selected ***Vanke*** as the case to study the governance problems of ***Vanke*** and the protection of the interests of small and medium shareholders under the situation of equity disputes. At the same time, the study further explored the advantages and disadvantages of the dispersed ownership structure, the long-term impact on the company’s development and the choice of the involved corporate governance methods under the current Chinese capital market conditions. This paper adopted the event research method and selected the period from June 2015 to June 2017 (24 months) as the observation period to analyze the market performance impact of ***Vanke*** in the equity disputes. At the same time, this paper also measured ***Vanke***’s individual stock rate of return (*R*_*it*_) and market rate of return (*R*_*mt*_), and calculated ***Vanke***’s normal rate of return [*E*(*R*_*i,t*_)], abnormal rate of return (*AR*_*i,t*_), and cumulative abnormal rate of return (*CAR*_*i*_) during different event windows ([−3,10]). ***Vanke***’s shareholding was too dispersed and the stock price had been sluggish for a long time, which had greatly reduced the acquisition difficulty and cost of ***Baoneng***, thus triggering the “barbarian invasion” of ***Baoneng***. In the struggle for control, whether it was ***Vanke***’s anti-takeover measures or ***China Resources***, ***Baoneng***, and ***Evergrande***’s competition for equity, their actions had harmed the interests of small and medium shareholders. The market supervision department was too lenient to supervise and punish the interests of small and medium shareholders, and opportunism made behaviors that infringe on the interests of others more reckless. However, small and medium shareholders cannot actively participate in the company’s management decision-making to safeguard their legitimate rights and interests, which intensifies the violations of all parties in the equity disputes, thus forming a vicious circle. Therefore, the protection of the interests of small and medium shareholders required the joint efforts and consciousness of regulators, small and medium shareholders, and acquirers.

## Introduction

China’s capital market has developed for more than 20 years and has achieved good results in financing and investment. However, when we talk about the stock market, we overemphasize its financing function, while ignoring that it is also a place for resource allocation and investment (Hayes and Lundholm, 1996; Hoberg and Phillips, 2016). Whether it was the stock market crash in 2007 or the market crash in 2015, the biggest losses were among small and medium shareholders (Bernard et al., 2006; Hughes et al., 2007; Armstrong et al., 2011; Dye and Hughes, 2018). If the stock market has become a place where “makers,” traders and listed companies wantonly plunder the interests of small and medium shareholders to satisfy their selfish desires, thus causing unbearable economic losses to small and medium shareholders, how can small and medium shareholders get involved in such a market? How can the capital market continue to move forward without the participation of small and medium shareholders (Christensen et al., 2010; Bertomeu et al., 2011; Corona and Lin, 2013; Caskey et al., 2015)? Therefore, from this perspective, protecting the interests of small and medium shareholders plays a vital role in the prosperity and development of China’s stock market.

The protection of the interests of small and medium shareholders involves the issue of principal–agent, and the principal–agent issue of management and shareholders has always been the basic problem in the corporate governance structure (Lemmon and Lins, 2003; Jessica, Weber, 2018). Among them, the ownership structure is one of the most core issues in corporate governance. An appropriate shareholding structure can not only help shareholders alleviate the “insider problem,” but also establish a monitoring mechanism for the behavior of large shareholders (Hodges et al., 2014). Seeking an equity structure suitable for corporate development should be the primary arrangement for corporate strategic development. Perfect corporate governance is not only an effective guarantee for modern companies to enhance their comprehensive competitiveness and performance capabilities, but also an important factor in attracting public investment investors (Robert, 1983). The combination of equity and governance can effectively help companies maximize corporate value and shareholder value.

The shareholding structure of Chinese listed companies is mostly highly concentrated, and the phenomenon of dominance caused by this is relatively common in China. Research showed that a highly concentrated ownership structure can solve the free-riding behavior of small and medium shareholders under a dispersed ownership structure. However, since other shareholders cannot effectively restrain large shareholders, absolute controlling shareholders can threaten the interests of small and medium shareholders by interfering with the management or colluding with them (Gebhardt et al., 2001; Francis et al., 2005; Fresard, 2010; Chen et al., 2014; Dhaliwal et al., 2016). A highly dispersed ownership structure can prevent the interests of major shareholders from infringing on the interests of small and medium shareholders, but it will also bring about the contradiction between the company’s public goods and the private costs of supervision.

This research can study the governance issues of ***Vanke*** and the protection of the interests of small and medium investors in the case of dispersed ownership structure. At the same time, this study can also further explore the advantages and disadvantages of dispersed ownership structure, the long-term impact on the company’s development, and the choice of corporate governance methods under the current Chinese capital market conditions. In this way, it can provide a reference for the corporate governance and equity setting of the same industry, as well as the protection of the interests of small and medium shareholders.

## Literature Review

### Ownership Structure and Agency

Most scholars generally believe that the centralized ownership structure can effectively prevent management from encroaching on shareholders for its own interests, and can reduce the occurrence of agency costs. Equity concentration will create a dominant phenomenon, which creates opportunities for large shareholders to hollow out small shareholders (Hail and Leuz, 2009; Christensen et al., 2010; Cheynel, 2013). The dispersed ownership structure can help to solve the problem that the company may have a dominant shareholder, but it may cause the insider control of the management.

The company’s shareholding structure is not in one-to-one correspondence with the internal and external mechanisms, that is, the dispersed shareholding does not mean that the external mechanism of the company must be good. Likewise, a high concentration of equity does not imply that internal mechanisms are functioning properly (Lang et al., 2004, 2012; Jeng and Pak, 2016). When both the internal mechanism and the external mechanism of the enterprise fail to deal with the principal–agent problem, the interest disputes between the large or small shareholder, the management, and the shareholder will exist within an enterprise at the same time.

### Ownership Structure and Corporate Governance

Some scholars believe that equity concentration is not conducive to the development of corporate performance. After the share-trading reform, the higher the company’s tradable shares, the greater the improvement in company performance (Hail and Leuz, 2009; Antoniou et al., 2016; Bertomeu and Cheynel, 2016). However, considering that most of the largest shareholders of domestic listed companies are sponsor shareholders, although the internal mechanism can reduce agency costs, it also creates convenience for the absolute controlling shareholder to control the management, conduct related transactions, occupy funds, and other behaviors that will reduce the company’s performance (Gode and Mohanram, 2003). Therefore, the concentration of the company’s ownership structure and the performance of the company are negatively correlated (Lambert et al., 2007).

Some scholars also pointed out that the more concentrated the company’s equity, the better the company’s performance. From the perspective of operation, the higher the company’s equity concentration, the better the company’s operating performance, and the two show a positive correlation (Easley et al., 2002; Botosan and Stanford, 2005; Papakroni, 2013). The performance of equity-concentrated companies is significantly better than that of equity-diversified companies, especially in an environment with immature capital markets, unsound laws, and insufficient investor protection. But at the same time, due to the existence of the absolute controlling shareholder (Ali et al., 2014), the interests of the small and medium shareholders have been damaged, so the centralized ownership structure is necessary to diversify the shareholding (Shleifer and Vishny, 1997).

### Ownership Structure and Protection of Interests of Small and Medium Shareholders

In a company with a high concentration of equity, the encroachment of small and medium shareholders is mainly manifested in the damage of the interests of small and medium shareholders by the controlling shareholder. Lemmon and Lins (2003) analyzed Asian listed companies in the economic crisis: due to the separation of control rights and cash flow rights caused by the pyramid ownership structure, the rights of controlling shareholders were expanded, which made large shareholders tend to encroach on the interests of small and medium shareholders, and the greater the degree of separation between the two, the more serious this phenomenon was (Minutiello and Tettamanzi, 2022).

In a dispersed ownership structure, this kind of infringement is often caused by management, which can be mitigated through equity incentives (Francis et al., 2008; Dhaliwal et al., 2011). When the external capital market is imperfect, the internal mechanism is imperfect, and the protection of investors is insufficient, the management will control the formulation and implementation of equity incentives (Kammoun and Djerry, 2021). This makes this incentive a way for management to gain vested interests rather than protect shareholder interests.

When the shareholding level of large shareholders is maintained between 35 and 63%, the proportion of large shareholders’ equity increases, and the private interests obtained by large shareholders out of small shareholders are more difficult to compensate for the loss of cash flow rights (Petersen, 2009; Suijs and Wielhouwer, 2019). Therefore, within this range, the increase of the large shareholder’s equity will have a positive effect on corporate performance. The high concentration of equity is actually a substitute for the lack of legal protection of shareholders’ interests. Therefore, from another perspective, highly concentrated equity is actually a protection for shareholders, but it is more focused on large shareholders (Chen, 2018; Li et al., 2021). The centralized ownership structure can prevent insider control and reduce agency costs, but it has a “tunnel effect.” Therefore, we should focus on how to protect the interests of small and medium shareholders for the prevailing dominance phenomenon in our country.

### Ownership Structure and Control Competition

It is widely believed that a dispersed ownership structure will invite hostile takeovers. However, in some special cases, shareholders within a company with a dispersed ownership structure may boycott the acquisition, which will be detrimental to the realization of the acquisition (Collins, 2011; Thomas, 2010). The equity alliance formed by different shareholders within the enterprise can resist the market’s acquisition of the company, and a solid alliance structure can never let the acquirer retreat or put the acquisition in a huge predicament (Cohen and Malkogianni, 2021.

The consensus view of most scholars is that, compared with companies with concentrated ownership, companies with dispersed ownership are more likely to compete for control rights. Suijs and Wielhouwer (2019) explained the relationship between ownership structure and control rights from the perspective of company value (Orhun, 2019). He established a company value model, and found that the contention for control can improve the value of a company with a dispersed ownership structure, but it is the opposite for a company with a centralized ownership structure. Shleifer and Vishny, 1997 believed that shareholders cannot effectively supervise management, which is prone to principal–agent problems, and the control market will introduce external large shareholders to supervise management, which is more likely to lead to control competition.

## Event Review

**FIGURE 1:**
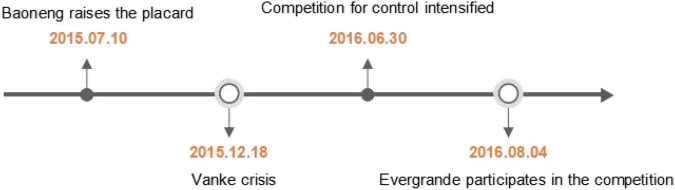
Timeline of the Vanke equity dispute.

In 1988, ***Vanke*** was formally established and completed the shareholding system reform; in the same year, it entered the real estate industry. So far, ***Vanke*** has completed the transformation from a single operation to a diversified operation. In 1991, ***Vanke*** was listed on the A-share market, becoming one of the earliest listed companies in China. As the largest residential development company in mainland China, ***Vanke*** has been included in several Forbes rankings, and has been rated as “China’s Most Responsive Real Estate Aircraft Carrier,” “100 Billion Aircraft Carrier,” and “Leader in Corporate Transformation.” By the end of 2015, ***Vanke*** had total assets of 611.3 billion yuan, net assets of 136.3 billion yuan, operating income of 195.5 billion yuan, and net profit of 18.1 billion yuan, an increase of 15.3% over the same period in 2014, and a return on net assets of 19.14% in the same year, still the largest real estate company in the industry. Since 2015, Vanke’s equity dispute has gradually attracted the attention of the public (Figure 1).

### Baoneng Raises the Placard

On July 10, 2015, ***Qianhai Life***, a subsidiary of ***Baoneng***, purchased 5% of ***Vanke*** stocks for the first time. On July 24, ***Jushenghua***, a concerted action person of ***Qianhai Life***, once again purchased the stocks of ***Vanke*** and became the second largest shareholder of ***Vanke*** with 10% of the stocks. On August 26, ***Baoneng*** once again increased its shareholding in ***Vanke*** by 5.04%, surpassing ***China Resources*** for the first time with a shareholding ratio of 15.04%, and became the largest shareholder of ***Vanke***. Subsequently, ***China Resources*** once again became the largest shareholder of ***Vanke*** with a 15.29% stake through two stock increases in August and September (Wang et al., 2019).

Since then, ***Baoneng*** and ***Vanke***’s equity dispute around ***Vanke*** has reached a fever pitch. On December 17, 2015, ***Baoneng*** once again became the largest shareholder of ***Vanke*** with a 23.52% stake. So far, ***Baoneng***’s shareholding ratio was close to the red line of the company’s acquisition, and ***Vanke*** was facing the biggest acquisition crisis since the “*Baoneng Battle*” in 1994 (Zhang, 2018). On the same day, ***WANG Shi***, the founder of ***Vanke***, publicly declared that the “*Battle of Wanke*” officially started.

### Vanke Crisis

On December 18, 2015, after the daily trading limit, the management of ***Vanke*** submitted a request to the China Securities Regulatory Commission to suspend trading on the grounds of asset restructuring and acquisition, and the trading of ***Vanke***’s A stocks and H stocks was suspended on the same day.

After that, ***Vanke*** united all parties to resist ***Baoneng***’s acquisition: on December 23, 2015, it sought ***Anbang*** as a concerted party. On March 3, 2016, ***China Resources*** pledged to support ***Vanke*** as always. On March 13, ***Vanke*** wanted to introduce ***Shenzhen Metro*** as its concerted action.

The situation took a dramatic turn: on March 17, 2016, after ***Vanke*** announced its cooperation with ***Shenzhen Metro***, ***China Resources*** suddenly raised a question to the regulatory authorities about ***Vanke***’s operational compliance. In June of the same year, the decision to purchase ***Shenzhen Metro*** assets was opposed by three directors of ***China Resources***. The next day, ***China Resources*** once again questioned the rationality of ***Vanke***’s restructuring plan for ***Shenzhen Metro***. On June 23, the two large shareholders of ***China Resources*** and ***Baoneng*** stated that they would jointly oppose the asset reorganization of ***Vanke*** and ***Shenzhen Metro***.

On June 24, 2016, ***China Resources*** once again became the largest shareholder of ***Vanke***, and then ***Baoneng*** requested the removal of seven directors including ***WANG Shi*** and ***YU Liang***, two independent directors, and three supervisors. At ***Vanke***’s annual shareholder meeting on June 27, shareholders focused on whether to remove 12 directors and high salaries for management.

### Intensifying Control Competition

On June 30, 2016, ***China Resources*** announced to the public that the company would not agree to the resolution to remove the board of directors of ***Vanke***. The reason was: the large-scale recall will have a serious adverse impact on ***Vanke***’s future medium and long-term development. The move once again sparked public discussion and controversy over ***Vanke***’s corporate governance.

On July 4, 2016, the first day of ***Vanke***’s resumption of trading, it encountered the limit down. The next day, ***Jushenghua*** once again purchased a total of 75 million ***Vanke*** A stocks. So far, ***Baoneng*** holds 24.97% of ***Vanke***’s total stocks (Venkatesh and Chiang, 2012). The frequent placards of the ***Baoneng*** were once again resisted by the management of ***Vanke***: on July 14, ***Vanke*** announced that it would jointly acquire a commercial real estate company with a mysterious partner. On July 18, ***Vanke*** reported to the China Securities Regulatory Commission, Shenzhen Stock Exchange and other regulatory agencies that ***Baoneng***’s asset management plan was suspected of irregularities, resulting in ***Baoneng***’s disclosure of nine asset management plans.

### Evergrande Participates in the Competition

On August 4, 2016, ***Evergrande*** purchased 517 million stocks of ***Vanke*** for 9.1 billion yuan, with a shareholding ratio of 4.68%. In the following 3 months, ***Evergrande*** continued to increase its holdings of ***Vanke*** by acquiring tradable stocks. As of November 29, the ***Vanke*** A stocks held by ***Evergrande*** accounted for 14.07% of ***Vanke***’s total stock capital, making it the company’s third largest shareholder, close to ***China Resources***, the second largest shareholder (Verrecchia and Weber, 2006).

Before that, ***Vanke***’s equity setting and management had been considered a model in the real estate industry. The occurrence of the control competition rights has attracted widespread attention from media public opinion and industry scholars, leading to a great discussion on corporate governance and equity setting. In addition, regulators and mainstream media, including many scholars, have called for all parties to pay attention to the protection of the interests of small and medium shareholders.

## Damage to the Interests of Small and Medium Shareholders

### Damage of Baoneng Acquisition to the Interests of Small and Medium Shareholders

#### Operational Turbulence Results

The adverse impact of the hostile takeover on ***Vanke***’s sales scale was shown in Table 1. ***Vanke***’s sales in July 2016 fell by 35% year-on-year, a much faster decline than the same period in 2015. In addition to the off-season sales, the reason for the rapid decline in performance is the struggle for control. Especially in June, ***Baoneng*** proposed to dismiss all the management, which forced the management center of ***Vanke*** to shift to the equity disputes, which seriously affected the normal operation.

**TABLE 1:** Sales scale of Vanke in July 2016.

	June 2016	July 2016	Chain growth rate (%)
Sales area (100 million square meters)	326.4	207.4	−36.40
Sales amount (100 million yuan)	424	274.4	−35.30

	**June 2015**	**July 2015**	**Chain growth rate (%)**

Sales area (100 million square meters)	195.5	181.5	−7.20
Sales amount (100 million yuan)	251.9	238.5	−5.30

*Data source: China Vanke Co., Ltd. 2016 Semi-Annual Report, 2016. http://www.cfi.net.cn/p20160821000814.html.*

The adverse impact of the hostile takeover on ***Vanke***’s operating income and profit is shown in Table 2. In the first half of 2016, the growth rate of ***Vanke***’s gross profit was far behind that of operating income. Compared with the same period in 2015, the profitability of enterprises has dropped significantly. From the perspective of the real estate industry alone, the interest rate of ***Vanke***’s real estate industry in 2016 also dropped by nearly three points compared with the same period in 2015.

**TABLE 2:** Profits of Vanke in the first half of 2016.

	January–June 2015	Year-on-year growth rate (%)	January–June 2016	Year-on-year growth rate (%)
Operating income (100 million yuan)	503.8	22.72	748.5	48.80
Operating profit (100 million yuan)	99.3	27.17	87.2	13.60

*Data source: China Vanke Co., Ltd. 2016 Semi-Annual Report, 2016. http://www.cfi.net.cn/p20160821000814.html.*

The competition for control caused by the hostile takeover has caused many unstable factors to the normal operation of ***Vanke***, which was contrary to the demands of the small and medium shareholders for the company’s stable operation, so that the investment returns of the majority of small and medium shareholders cannot be effectively guaranteed.

#### Violent Stock Price Fluctuations Leading to Irrational Investment

This paper selected the stock price within the event window ([−3,10]) to measure the market’s reaction to the ***Vanke*** equity disputes. This paper measured ***Vanke***’s individual stock rate of return *R*_*i,t*_ and market rate of return *R*_*m,t*_, and calculates ***Vanke***’s normal rate of return *E*(*R*_*i,t*_), Abnormal Return *AR*_*i,t*_ and Cumulative Abnormal Return *CAR*_*i*_ in different event windows (Bertomeu, 2015; Hua et al., 2018).

i) Calculating the coefficients α and β.

$$Ri,t=\alpha_{i}+\beta_{i}R_{m,t}+\varepsilon t$$

*R*_*i,t*_ refers to the actual rate of return of a stock on day *t*, the specific arithmetic formula is

*R*_*i*,*t*_ = (*P*_*i*,*t*_−*P*_*i*,*t*−1_)/*P*_*i*,*t*−1_, and *P*_*i,t*_ is the closing price of stock *i* on day *t*; *R*_*m,t*_ is the market rate of return, and its specific calculation method is

**FIGURE 2:**
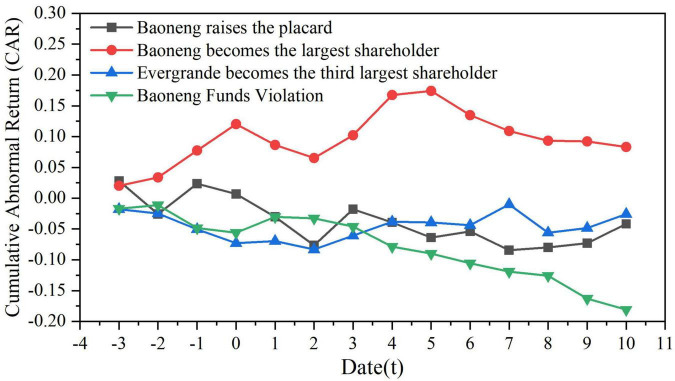
Cumulative abnormal return trends in each event window for control rights. Data source: Derived from the CAR value calculated from the stock price (Supplementary Table 1).

*R*_*m*,*t*_ = (*I*_*t*_−*I*_*t*−1_)/*I*_*t*−1_. *I*_*t*_ in this paper is the Shenzhen stock index on the *t* day. In addition, *α_*i*_* and *β_*i*_* are parameters to be solved, and *ε_*t*_* is a random error term.

ii) Calculating the normal rate of return *E*(*R*_*i,t*_).

$$E\left(R_{i,t}\right)=\alpha_{i}+\beta_{i}R_{m,t}+\varepsilon t$$

iii) Calculating Abnormal Return (*AR*_*i,t*_) and Cumulative Abnormal Return (*CAR*_*i*_).

$$AR_{i,t}=R_{i,t}-E\left(R_{i,t}\right),CAR_{i}=\sum AR_{i,t}\left(t=t_{1,}\ldots \ldots ,t_{2}\right)$$

iv) Performing a significance test on the Cumulative Abnormal Return (*CAR*), and the *CAR* trend chart in Figure 2 is obtained.

The CAR value of ***Baoneng*** showed an overall downward trend and was negative, indicating that the ***Vanke*** equity dispute had greatly reduced the market’s expectations for ***Vanke***, and the excess rate of return had reached −3.6 and −4.6%. The management’s factional dividend plan led to a brief increase in the CAR value (*t* = 3), but then it fell again, and the CAR value began to gradually rise to 0 again at *t* = 7. After ***Baoneng*** became the first shareholder of ***Vanke***, the CAR value decreased at *t* = 1 and *t* = 2, and the excess returns were −3.6 and −1.4%, respectively; it started to pick up in the next 3 days and then decreased again in the last 5 days of the window.

A negative excess rate of return indicates that the interests of small and medium shareholders have been violated. At the same time, violent stock price fluctuations increase the capital risk of the acquirer, which may further damage the interests of small and medium shareholders after the acquisition is completed. Considering that ***Baoneng*** acquired ***Vanke*** this time through a large number of asset management products, leveraged financing and stock pledges, once ***Baoneng*** completes the acquisition, in order to quickly make up for capital costs and risks, the acquirer is likely to harm the interests of small and medium shareholders by transferring and hollowing out ***Vanke***’s high-quality assets.

#### Bankruptcy of Stock Repurchase Plan

**FIGURE 3:**
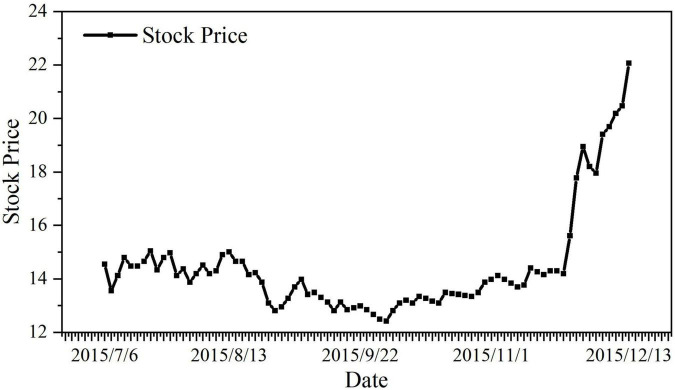
Stock price chart after Vanke announced stock repurchase plan. Data source: Derived from the CAR value calculated from the stock price (Supplementary Table 1).

Stock repurchase can bring about a steady rise in stock price, improve the company’s shareholding structure, and improve corporate governance, which is conducive to the company’s future stable development and avoidance of hostile takeovers. Therefore, ***Vanke*** released the company’s stock repurchase plan on July 6. However, according to Figure 3, the stock price of ***Vanke*** has been higher than its actual highest price of 13.16 yuan for a long time; judging from the repurchase results, the highest price of ***Vanke***’s repurchase was 13.16, and the repurchase plan was finally executed only 160 million yuan, only 1.6% of the expected. ***Vanke***’s repurchase plan was nearly bankrupt.

In the short term, ***Baoneng***’s hostile takeover has actually caused violent fluctuations in the stock price, making it difficult for small and medium shareholders to enjoy capital gains in the secondary market (Zheng et al., 2021). In the long term, the bankruptcy of the repurchase plan has caused ***Vanke*** to fail to improve its own equity structure as scheduled, and it has fallen into a long-term chaotic and deformed struggle for control, which has seriously damaged the long-term interests of small and medium shareholders.

#### Management Crisis

In order to ensure the completion of the acquisition and obtain the board position of ***Vanke***, ***Baoneng*** proposed to remove the senior members of ***Vanke*** as the largest shareholder, resulting in a significant drop in the company’s sales scale and revenue in July compared with the same period in the past. Combining Tables 1, 2, the acquirer abused the authority of the largest shareholder in order to realize its own interests, causing serious turmoil in ***Vanke***’s management and a significant decline in its performance. The stability of the company’s operation will be greatly reduced, which will make the market’s expectations for the company fall rapidly, which is not conducive to the company’s long-term development, thereby causing damage to the interests of small and medium shareholders.

### Damage of Evergrande’s Intervention to the Interests of Small and Medium Shareholders

#### Violation of the Principle of Information Disclosure

The information disclosure system was originally designed to alleviate the disadvantage of the natural information asymmetry of small and medium shareholders (Darrough, 1993), but in order to reduce the acquisition cost, ***Evergrande*** not only failed to publish transaction information in a timely manner, but also spread false information everywhere (Zhou, 2019). The above practices seriously violated the requirements of the regulators for information disclosure of listed companies, and disrupted the order of the securities market and the stability of stock prices. The majority of small and medium shareholders are unable to achieve investment returns amid stock price fluctuations, and ***Evergrande***’s attempt to gain private ownership by manipulating stock prices is extremely unfair to small and medium shareholders.

#### The Rising Risk of the Company in the Future

**FIGURE 4:**
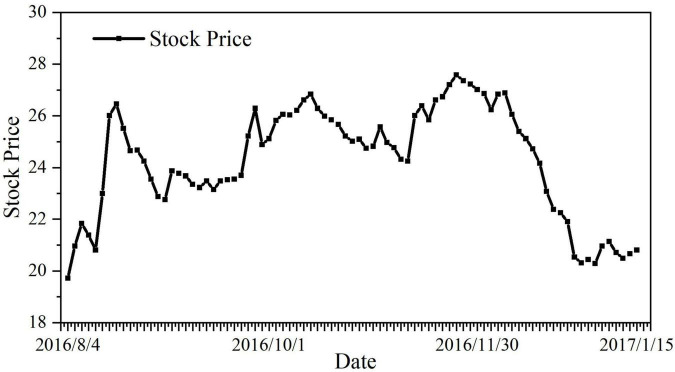
Vanke’s stock price chart after Evergrande raised the placard. Data source: http://stock.hexun.com/2016-08-04/185332058.html.

Figure 4 is the stock price chart of ***Vanke*** after ***Evergrande*** raised its placard. It can be found from the line chart that the increase of competing parties will increase the instability of the company’s stock price in the secondary market. Considering that the previous hostile takeover by ***Baoneng*** has led to the violent fluctuation of ***Vanke***’s share price, ***Evergrande***, as a new contender, will further aggravate this instability, which is a fatal blow to the interests of ***Vanke***’s small and medium shareholders.

**FIGURE 5:**
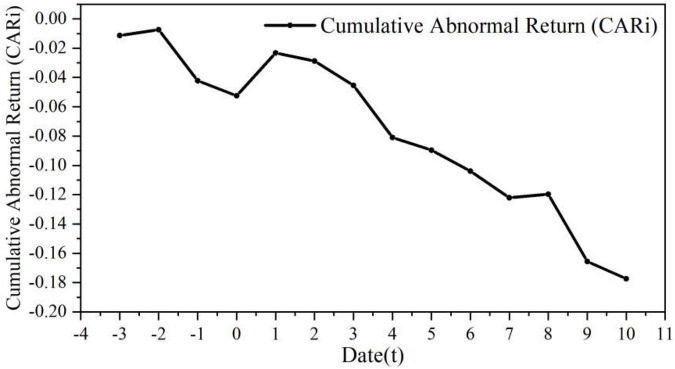
Changes in CAR of Evergrande becoming the third largest shareholder. Data source: Derived from the CAR value calculated from the stock price (Supplementary Table 1).

According to Figure 5, the trend of excess return in the above chart shows that the capital market has responded more quickly and farther to the event that ***Evergrande*** became the third largest shareholder, which proves from a market perspective that this event is not conducive to the interests of small and medium shareholders.

***Evergrande*** becoming the third-largest shareholder has investors uncertain about the future of the battle for control of ***Vanke***. What’s more serious is that due to ***Evergrande*** becoming the third largest shareholder, the outstanding shares of ***Vanke***’s A-shares are only 14.65%. Too few tradable shares indicate that ***Vanke***’s stock is likely to have become a “zhuanggu,” which will turn it into a profit tool for marketers to manipulate stock prices and trading volume. This will cause extremely serious damage to the trading rights and interests of small and medium shareholders.

### Damage to the Interests of Small and Medium Shareholders by the Management of Vanke

#### Hastily Introduced *Shenzhen Metro* to Dilute the Stocks Held by Small and Medium Shareholders

Management deliberately dilutes stocks. According to Table 3, it is found that the scale of ***Shenzhen Metro***’s assets is much higher than that of the same industry, but the level of operating income, net profit, and return on assets is much lower than the industry value. As the target of ***Vanke***’s intention to purchase, ***Shenzhen Metro*** is not only in average profitability, but also considering the long payback period of the subway project, and it is difficult to achieve income in the short term, the acquisition of such a company will drag down ***Vanke***’s previous excellent operating performance and profitability.

**TABLE 3:** Comparison of major financial indicators between Shenzhen Metro and the industry.

	Assets (100 million yuan)	Operating income (100 million yuan)	Net profit (100 million yuan)	Roe
Shenzhen Metro	2403.97	5.18	0.52	0.00035
Industry	245.98	8.48	0.77	0.22

*Data source: Proposal for Issuing Shares to Purchase Assets and Related Party Transactions, 2016. https://kuaixun.cngold.org/c/2016-06-18/c360495.html.*

Management overvalued acquired assets. According to Table 4, it is found that the valuation time of ***Shenzhen Metro*** and ***Vanke***’s major assets of ***Qianhai Life*** is only 20 days apart, but the valuation difference between the two is 22 billion yuan. The huge increase in a short period of time is suspected of being overvalued. According to Table 5, the shareholding ratio of ***Baoneng*** and ***China Resources*** decreased by 20.6% after the introduction of ***Shenzhen Metro***. Since the “poison plan” was used to dilute the ***Vanke*** stocks held by ***Baoneng*** and thus protect the management’s control, the biggest beneficiaries of the restructuring are obviously the management of ***Shenzhen Metro*** and ***Vanke***.

**TABLE 4:** Changes in the valuation of major assets of Qianhai Life.

Valuer	Shenzhen Metro	Vanke
Valuation (100 million yuan)	235.896	455.74
Valuation date	2016.5.26	2016.6.17

*Data source: Proposal for Issuing Shares to Purchase Assets and Related Party Transactions, 2016. https://kuaixun.cngold.org/c/2016-06-18/c360495.html.*

**TABLE 5:** Changes in Vanke’s shareholding structure after the introduction of Shenzhen Metro.

Shareholder	Shareholding ratio before additional issuance	Shareholding ratio after additional issuance	Ranking	Change in shareholding
*Shenzhen Metro*	0	20.65%	1	N/A
*Baoneng*	24.29%	19.27%	2	20.60%
*China Resources*	15.24%	12.10%	3	20.60%

*Data source: Proposal for Issuing Shares to Purchase Assets and Related Party Transactions, 2016. https://kuaixun.cngold.org/c/2016-06-18/c360495.html.*

The management of ***Vanke*** bypassed the board of directors and ignored the interests of all shareholders and insisted on introducing ***Shenzhen Metro***. In fact, it shows that there is a serious risk of insider control in ***Vanke***, and the interests of small and medium shareholders are obviously not effectively protected.

#### Restricting the Free Trading Rights of Small and Medium Shareholders

**FIGURE 6:**
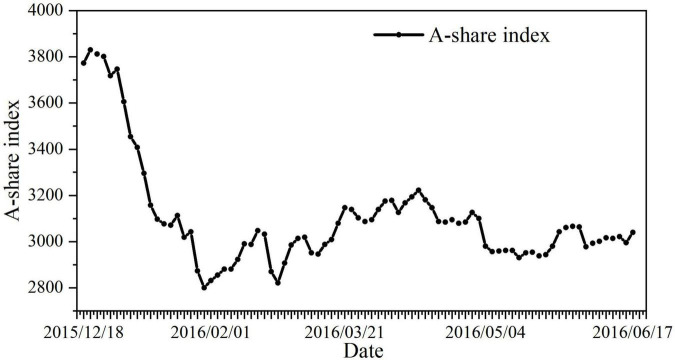
Trend chart of the A-share index during the suspension period of Vanke. Data source: Trend chart of the A-share index during the suspension period of Vanke. https://finance.eastmoney.com/news/1354,20160704638722937.html.

Before the suspension of ***Vanke***’s trading, the company’s 17 executives promptly emptied the company’s stocks with a total market value of nearly 30 million yuan. Their timing of the transaction is so accurate that it makes people suspect that it is suspected of leaking confidential information. According to Figure 6, the Shanghai Composite Index fell from 3600 to 2900 during the suspension period. Due to ***Baoneng***’s aggressive acquisitions, ***Vanke***’s stock price was already inflated before the suspension, which made ***Vanke***’s small and medium shareholders miss the best time to stop losses and exit the market, and their trading freedom was severely restricted. After the resumption of ***Vanke***’s trading, the stock price plummeted, and small and medium shareholders had to bear huge losses for the reckless and arbitrary management of the management.

### Damage to the Interests of the Small and Medium Shareholders Themselves

#### Lack of Judgment Leading to Irrational Investment

Due to the intervention of ***Baoneng*** and ***Evergrande***, there have been many cases of investors “chasing up and down.” The dispute over control has caused the instability of ***Vanke***’s stock price, coupled with the inability to arbitrage or stop losses in a timely manner, making small and medium shareholders “locked up” when the stock price is high, and ultimately causing damage to their own interests. Small and medium shareholders lack short-term capital technology and information channels. Compared with the fluke mentality of “making a lot of money,” it should be the attitude of small and medium shareholders not to rashly increase their holdings and stop losses in a timely manner.

#### Ignoring the Interests of Small and Medium Shareholders

***Vanke***, as a typical company with highly dispersed equity, holds a shareholding ratio of 65% in ***Vanke*** by small and medium shareholders. After ***Baoneng*** became the largest shareholder of ***Vanke***, the proportion of small and medium shareholders is still close to 50%. It can be said that the small and medium shareholders are the largest shareholders of ***Vanke*** in the true sense (Li et al., 2018).

In the dispute over control, any decision made by ***Vanke***’s management did not reflect the opinions of small and medium shareholders. On the contrary, whether it is the introduction of ***Shenzhen Metro*** or the hasty suspension of trading, every measure taken by the management to counter ***Baoneng*** has seriously damaged the interests of small and medium shareholders. The enthusiasm of small and medium shareholders to participate in the decision-making of company affairs is not high, and the existence of free-rider psychology makes it difficult for the company to make long-term decisions in line with the interests of small and medium shareholders.

## Reasons for the Damage to the Interests of Small and Medium Shareholders

### Unreasonable Equity Setting

From the perspective of the company’s equity protection, ***Vanke*** does not have a “partnership system” similar to ***Alibaba*** to ensure that the founder team can stably control the company. The founder ***WANG Shi*** tried to restrict ***Baoneng***’s acquisition by introducing ***Shenzhen Metro***, but was unexpectedly opposed by the “former owner” ***China Resources***. The prolonged suspension and struggle to deal with acquisitions have had a severe negative impact on ***Vanke***’s performance.

Various deeds in the equity disputes showed that ***Vanke***’s ownership structure was difficult to stabilize the company’s control, and at the same time, it cannot effectively reduce the adverse impact of hostile takeovers on the company’s long-term development. In fact, it was precisely because the equity setting was unreasonable that it led to the competition for its control rights, which ultimately damaged the interests of small and medium shareholders.

### Insider Control

Insider control is the biggest flaw in ***Vanke***’s corporate governance. The founder ***WANG Shi*** has a huge responsibility in the company’s operation and has played a pivotal role in ***Vanke***’s development and growth into the world’s best real estate developer. But in fact, ***China Resources***, as the largest shareholder, rarely participates in the company’s operations, and has not achieved real control.

In Table 6, ***Guoxin Jinpeng* No. 1** was a business partner system launched by ***Vanke***’s management in 2014, that is, ***Vanke***’s internal employees hold shares. ***Vanke*** executives represented by ***WANG Shi*** and ***YU Liang*** hardly hold the company’s stock, but they had long held the actual control of the company. Their decision-making in the battle for control was even more deviated from the interests of shareholders, and even in order to maintain their control, they used actions such as suppressing the stock price, hasty suspension of trading, and reorganization of shares. They had completely disregarded the interests of small and medium shareholders.

**TABLE 6:** Top 10 shareholders of Vanke before June 30, 2015.

	Name	Shareholding ratio (%)
1	China Resources Co., Ltd.	14.89
2	HKSCC Nominees Limited	11.90
3	Guoxin Jinpeng No. 1 Collective Asset Management Plan	4.14
4	GIC Private Limited	1.38
5	Liu Yuansheng	1.21
6	Merrill Lynch International	1.12
7	China Life – Personal Dividends	0.87
8	Vanke Trade Union Committee	0.61
9	China Life – General Insurance Products	0.57
10	UBS AG	0.54

*Data source: Ranking of the top 10 shareholders of Vanke on June 30, 2015. https://km.newhouse.fang.com/2016-06-27/21751733.htm.*

### Irrationality of Small and Medium Shareholders

#### Internal Reasons

The psychology of chasing up and down. In the struggle for control, ***Baoneng*** acquired a large number of tradable shares in the secondary market, which led to the rise of ***Vanke***’s share price, which triggered the psychology of small and medium shareholders chasing the rise and selling the fall. ***Baoneng*** used leveraged funds to leverage ***Vanke***, which was more speculative than investment. Small and medium shareholders blindly follow the trend and hope to get rich overnight, so they rush to buy ***Vanke*** stocks. The subsequent slump in stock prices and the suspension of trading for more than half a year not only caused small and medium investors to suffer unbearable losses, but also damaged their right to free trading.

#### External Reasons

All parties involved in the competition are suspected of various violations. A variety of violations such as insider trading, information disclosure violations, and stock price manipulation occurred in this control competition, which seriously affected the stability and fairness of the securities market. This is extremely detrimental to the protection of the interests of small and medium shareholders.

### Lack of Regulatory System

First, the enforcement of the information disclosure system is insufficient. For example, ***Evergrande*** did not comply with the truthfulness, accuracy, and completeness of the disclosed information when purchasing stocks of ***Vanke***. For another example, ***Vanke***’s forced trading suspension was suspected of insider trading, and the management did not comply with the fairness principle in the information disclosure system.

Second, the punishment is not strong. The various behaviors of all parties in this control right struggle have damaged the legitimate rights and interests of small and medium shareholders. However, the regulatory authorities did not take appropriate measures against the relevant personnel. This is more of an extrajudicial favor to the parties involved in the battle for control. Whether it was the management’s hasty suspension or the acquirer’s abuse of information disclosure, they were only criticized by the regulatory authorities without further disciplinary measures.

Finally, the litigation system is flawed. In this competition for control rights, in the face of the company’s management’s many reckless behaviors, the small and medium shareholders have no right to appeal for protection when their own rights and interests have been violated.

### Defects in the Independent Director System

According to Table 7, as of June 30, 2015, the board of directors of ***Vanke*** consisted of 11 members, including 4 independent directors and 7 directors.

**TABLE 7:** List of Vanke board of directors.

Name	Position	Affiliated company	Name	Position
*WANG Shi*	Chairman of the Board	Vanke	*ZHANG Liping*	Independent director
*WANG Wenjin*	Director	Vanke	*HAI Wen*	Independent director
*YU Liang*	Director	Vanke	*HUA Sheng*	Independent director
*SUN Jianyi*	Director	Ping An Bank	*LUO Junmei*	Independent director
*WEI Bin*	Director	China Resources		
*CHEN Ying*	Director	China Resources		
*QIAO Shipo*	Director	China Resources		

*Data source: https://topic.eastmoney.com/wank2015/.*

First, the independence of independent directors was questioned. On June 18, 2016, in ***Vanke***’s proposal to introduce ***Shenzhen Metro***, ***ZHANG Liping*** had a relationship with the company’s production and operation, but served as an independent director of the company. The economics and independence of independent directors had been severely affected. Obviously, ***ZHANG Liping*** should not continue to serve as an independent director of ***Vanke***.

Second, independent directors were suspected of revealing company secrets. In addition, during equity disputes, independent director ***HUA Sheng*** repeatedly published highly targeted and offensive remarks in the public media, including criticism of the company’s management, opposition to large shareholders and the details of the board’s decision-making. This was not in line with the code of conduct for the position in the first place, and its compliance legitimacy had been questioned.

Finally, the independent directors were suspected of not fully performing their duties. In this equity disputes, ***WANG Shi*** publicly doubted the rationality of ***Baoneng*** becoming the largest shareholder of ***Vanke*** in the media, and then ***HUA Sheng*** began to question the motive and ability of ***Baoneng***’s acquisition in the article. However, the independent directors representing the interests of small and medium shareholders did not raise any different opinions. This had made it hard to believe that the independent director system of ***Vanke*** really fully performs its duties.

## Discussion

### For Regulators

#### Improving the Information Disclosure System

The information disclosure of listed companies is an important means to make up for the information asymmetry in the securities market and meet the information requirements of the majority of investors. Therefore, for individuals or collectives who do not use or even abuse information disclosure tools correctly, the regulatory authorities should severely punish and strengthen supervision in this regard.

#### Improving the Accountability Mechanism for Damage to the Interests of Small and Medium Shareholders

The important reason why it is difficult to effectively protect the interests of small and medium shareholders is that China currently lacks an effective mechanism for small and medium shareholders to hold listed companies accountable, so that the illegal cost of all parties infringing the interests of small and medium shareholders is extremely low (Ball et al., 2016). Therefore, market regulators should promptly introduce civil accountability systems related to abusive information disclosure, insider trading, and stock price manipulation, and compel violators to make economic compensation for small and medium shareholders whose interests have been damaged from the perspective of laws and regulations. It can not only play a warning role, but also effectively curb the recurrence of violations of relevant regulations (Jonathan Lewellen, 2015).

### For Listed Companies

#### Allocating Shareholder Power Reasonably

To improve the shareholding structure, it is necessary to ensure the control and balance of shareholders. When improving the company’s equity structure, listed companies should pay attention to ensuring that the company’s shareholders can effectively control the company’s control rights and prevent the recurrence of equity disputes. In order to protect the interests of small and medium shareholders, the improvement of the shareholding structure should also pay attention to the ability to effectively check and balance other shareholders, and at the same time to supervise the management’s business decisions.

#### Strengthening the Market Value Management of Listed Companies

From the perspective of protecting the interests of small and medium shareholders, market value management focusing on the long-term development of the company and building investor relations can not only reflect the price discovery function of the capital market, but also help protect the interests of small and medium investors.

### For Small and Medium Shareholders

Small and medium shareholders need to be rational in the secondary market. When investing, small and medium shareholders should avoid following the trend, “fighting the luck” and blindly listening to gossip, maintain due vigilance, and carefully screen the asset status and future value of listed companies (Valta, 2012). Small and medium shareholders should make rational investments according to their own risk appetite and actual economic level, and must not have the mentality of “a huge profit” and “get rich overnight,” so as to avoid unnecessary losses to themselves.

Small and medium shareholders should also actively participate in the management of the company. At this stage, online voting at the general meeting of shareholders of listed companies has become popular (Li, 2010). Only by actively and responsibly exercising their legal rights and actively participating in the company’s business decisions can small and medium shareholders effectively safeguard their own interests.

### For Acquirers

Acquirers should spontaneously form an attitude of maintaining market order and the interests of small and medium shareholders. Protecting the interests of small and medium shareholders requires the self-consciousness of the acquirer. Acquirers cannot only consider the results of business competition and ignore the legitimate rights and interests of small and medium investors (Berger et al., 2012). In the competition for control rights, we should consciously abide by market rules, and there should not be behaviors such as manipulation of stock prices, insider trading, and abuse of information disclosure tools that disrupt market order and investor confidence.

## Conclusion

This paper took the equity dispute of ***Vanke*** as the background, and mainly studied the damage to the interests of small and medium shareholders in the equity dispute. This paper mainly drew the following conclusions:

First of all, the highly dispersed ownership structure is the main reason why ***Vanke*** has encountered equity dispute. ***Vanke***’s shareholding is too dispersed and the stock price has been sluggish for a long time, which has greatly reduced the acquisition difficulty and cost of ***Baoneng***, thus triggering ***Baoneng***’s brutal invasion. The dispersed ownership structure also led to ***Vanke***’s long-term insider control of the management. There was a backlash from management to defend its control when the company faced a hostile takeover. The above two factors eventually led to the occurrence of the ***Vanke*** equity dispute.

Second, in the struggle for control, neither the management, major shareholders nor acquirers take into account the interests of small and medium shareholders. Whether it is the anti-takeover measures taken by the management of ***Vanke***, or the equity dispute by ***China Resources***, ***Baoneng***, and ***Evergrande***, their actions have actually caused damage to the interests of small and medium shareholders.

Finally, the protection of the interests of small and medium shareholders is imminent. The regulatory authorities are too lenient to supervise and punish all parties that harm the interests of small and medium shareholders, and the existence of opportunism makes them more reckless with their actions that infringe on the interests of others. However, the small and medium shareholders lack effective ways to safeguard their legitimate rights and interests by participating in the company’s management decision-making, which intensifies the violations of all parties in the equity dispute, thus forming a vicious circle.

At present, my country’s regulatory agencies, listed companies or small and medium shareholders themselves lack substantial measures to protect the interests of small and medium shareholders. In the subsequent research process, this paper hopes to use other research methods to conduct a more comprehensive and systematic analysis of the large sample data of the capital market, so as to draw universal conclusions.

## Note

China Vanke Co., Ltd. is referred to as ***Vanke***.
Shenzhen Baoneng Investment Group Co., Ltd. is referred to as ***Baoneng***.
China Resources Group is referred to as ***China Resources***.
Evergrande Real Estate Group Co., Ltd. is referred to as ***Evergrande***.
Qianhai Life Insurance Co., Ltd. is referred to as ***Qianhai Life***.
Shenzhen Jushenghua Co., Ltd. is referred to as ***Jushenghua***.
Anbang Insurance Group Co., Ltd. is referred to as ***Anbang***.
Shenzhen Metro Group Co., Ltd. is referred to as ***Shenzhen Metro***.
Company names are in bold italics, such as ***Baoneng***.
People’s names are in bold italics, and all letters of the last name are capitalized, such as ***WANG Shi***.

## Data Availability Statement

The original contributions presented in the study are included in the article/Supplementary Material, further inquiries can be directed to the corresponding author.

## Author Contributions

CH: writing – review and editing, data curation, and formal analysis. WS: conceptualization and writing – review and editing. TO: investigation, formal analysis, and supervision. WL: resources, visualization, and supervision. BZ: resources, formal analysis, visualization, and supervision. All authors contributed to the article and approved the submitted version.

## Conflict of Interest

The authors declare that the research was conducted in the absence of any commercial or financial relationships that could be construed as a potential conflict of interest.

## Publisher’s Note

All claims expressed in this article are solely those of the authors and do not necessarily represent those of their affiliated organizations, or those of the publisher, the editors and the reviewers. Any product that may be evaluated in this article, or claim that may be made by its manufacturer, is not guaranteed or endorsed by the publisher.
