# Supplementary material for: Analysis of Equity Disputes in Listed Companies With Dispersed Ownership Structure and Protection of Small and Medium Shareholders’ Interests
Source: Front Psychol. 2022 May 20;13:857585. doi: 10.3389/fpsyg.2022.857585 (PMC9163785; doi:10.3389/fpsyg.2022.857585)
Supplement: Supplementary file 1 [file Table_1.DOC]

**Calculation details of CAR value in each event window period**

**Schedule 1. Cumulative Excess Return Rate of "Baoneng's First Acquisition of Vanke"**

| ***t***= | Trading Day | Market Return Rate | Normal Return Rate | Real Return Rate | Excess Return Rate | CAR |
| --- | --- | --- | --- | --- | --- | --- |
| -3 | 2015/7/7 | -0.058 | -0.0525 | -0.0228 | 0.0296 | 0.0296 |
| -2 | 2015/7/8 | -0.0294 | -0.0262 | -0.088 | -0.0619 | -0.0322 |
| -1 | 2015/7/9 | 0.0425 | 0.04 | 0.0973 | 0.0573 | 0.025 |
| 0 | 2015/7/10 | 0.0459 | 0.0431 | 0.0289 | -0.0142 | 0.0108 |
| 1 | 2015/7/13 | 0.0478 | 0.0449 | 0.0087 | -0.0362 | -0.0254 |
| 2 | 2015/7/14 | 0.0091 | 0.0092 | -0.0364 | -0.0457 | -0.0711 |
| 3 | 2015/7/15 | -0.0468 | -0.0422 | 0.011 | 0.0532 | -0.0179 |
| 4 | 2015/7/16 | 0.0186 | 0.018 | -0.0007 | -0.0186 | -0.0365 |
| 5 | 2015/7/17 | 0.0524 | 0.0491 | 0.0252 | -0.0239 | -0.0604 |
| 6 | 2015/7/20 | 0.0152 | 0.0149 | 0.0186 | 0.0037 | -0.0567 |
| 7 | 2015/7/21 | 0.0086 | 0.0088 | -0.0222 | -0.0309 | -0.0876 |
| 8 | 2015/7/22 | 0.0076 | 0.0079 | 0.0131 | 0.0052 | -0.0824 |
| 9 | 2015/7/23 | 0.0252 | 0.0241 | 0.0313 | 0.0072 | -0.0752 |
| 10 | 2015/7/24 | -0.0172 | -0.0149 | 0.0165 | 0.0314 | -0.0438 |

**Schedule 2. Cumulative Excess Return of "Baoneng Became the Largest Shareholder for the First Time"**

| ***t***= | Trading Day | Market Return Rate | Normal Return Rate | Real Return Rate | Excess Return Rate | CAR |
| --- | --- | --- | --- | --- | --- | --- |
| -3 | 2015/8/21 | -0.0542 | -0.0443 | -0.0259 | 0.0184 | 0.0184 |
| -2 | 2015/8/24 | -0.0783 | -0.0643 | -0.0518 | 0.0125 | 0.031 |
| -1 | 2015/8/25 | -0.0704 | -0.0578 | -0.0144 | 0.0434 | 0.0743 |
| 0 | 2015/8/26 | -0.0292 | -0.0236 | 0.0192 | 0.0428 | 0.1172 |
| 1 | 2015/8/27 | 0.0358 | 0.0304 | -0.0053 | -0.0357 | 0.0815 |
| 2 | 2015/8/28 | 0.0532 | 0.0448 | 0.0303 | -0.0145 | 0.067 |
| 3 | 2015/8/31 | -0.0232 | -0.0186 | 0.0162 | 0.0348 | 0.1018 |
| 4 | 2015/9/1 | -0.0367 | -0.0298 | 0.0312 | 0.0609 | 0.1627 |
| 5 | 2015/9/2 | -0.0106 | -0.0081 | -0.0007 | 0.0074 | 0.1701 |
| 6 | 2015/9/7 | -0.0063 | -0.0045 | -0.0436 | -0.0391 | 0.1311 |
| 7 | 2015/9/8 | 0.0329 | 0.028 | 0.0059 | -0.0221 | 0.109 |
| 8 | 2015/9/9 | 0.0291 | 0.0248 | 0.0058 | -0.0189 | 0.0901 |
| 9 | 2015/9/10 | -0.0184 | -0.0146 | -0.016 | -0.0014 | 0.0887 |
| 10 | 2015/9/11 | 0.0037 | 0.0038 | -0.0052 | -0.0089 | 0.0797 |

**Schedule 3. Cumulative Excess Return of “Evergrande Becoming the Third Largest Shareholder”**

| ***t***= | Trading Day | Market Return Rate | Normal Return Rate | Real Return Rate | Excess Return Rate | CAR |
| --- | --- | --- | --- | --- | --- | --- |
| -3 | 2016/11/24 | -0.0006 | 0.0029 | -0.0107 | -0.0136 | -0.0136 |
| -2 | 2016/11/25 | 0.0063 | 0.0063 | 0.01 | 0.0037 | -0.0099 |
| -1 | 2016/11/28 | 0.0029 | 0.0047 | -0.0301 | -0.0348 | -0.0447 |
| 0 | 2016/11/29 | -0.0031 | 0.0017 | -0.0083 | -0.01 | -0.0547 |
| 1 | 2016/11/30 | -0.002 | 0.0022 | 0.0313 | 0.0292 | -0.0255 |
| 2 | 2016/12/1 | 0.0069 | 0.0066 | -0.0004 | -0.007 | -0.0326 |
| 3 | 2016/12/2 | -0.0158 | -0.0047 | -0.0193 | -0.0146 | -0.0471 |
| 4 | 2016/12/5 | -0.0118 | -0.0027 | -0.0359 | -0.0332 | -0.0804 |
| 5 | 2016/12/6 | -0.0004 | 0.003 | -0.0047 | -0.0077 | -0.088 |
| 6 | 2016/12/7 | 0.007 | 0.0067 | -0.0095 | -0.0162 | -0.1042 |
| 7 | 2016/12/8 | -0.004 | 0.0012 | -0.0159 | -0.0171 | -0.1213 |
| 8 | 2016/12/9 | -0.0021 | 0.0022 | 0.0028 | 0.0007 | -0.1207 |
| 9 | 2016/12/12 | -0.0451 | -0.0194 | -0.0625 | -0.0431 | -0.1638 |
| 10 | 2016/12/13 | 0.0029 | 0.0046 | -0.0086 | -0.0132 | -0.177 |

**Schedule 4. Excess Return Rate of “Vanke Report Baoneng Asset Management Plan”**

| ***t***= | Trading Day | Market Return Rate | Normal Return Rate | Real Return Rate | Excess Return Rate | CAR |
| --- | --- | --- | --- | --- | --- | --- |
| -3 | 2016/7/14 | 0.0001 | 0.0017 | -0.0197 | -0.0214 | -0.0214 |
| -2 | 2016/7/15 | -0.0028 | 0.0002 | -0.0039 | -0.0041 | -0.0254 |
| -1 | 2016/7/18 | -0.0057 | -0.0014 | -0.0257 | -0.0244 | -0.0498 |
| 0 | 2016/7/19 | 0.0015 | 0.0025 | -0.0184 | -0.0208 | -0.0706 |
| 1 | 2016/7/20 | -0.0017 | 0.0008 | -0.0018 | -0.0025 | -0.0731 |
| 2 | 2016/7/21 | 0.0025 | 0.003 | -0.0035 | -0.0066 | -0.0797 |
| 3 | 2016/7/22 | -0.0072 | -0.0022 | 0.0217 | 0.0239 | -0.0558 |
| 4 | 2016/7/25 | 0.0009 | 0.0021 | 0.0178 | 0.0157 | -0.0401 |
| 5 | 2016/7/26 | 0.0125 | 0.0084 | 0.0045 | -0.0039 | -0.0439 |
| 6 | 2016/7/27 | -0.0411 | -0.0204 | -0.0202 | 0.0001 | -0.0438 |
| 7 | 2016/7/28 | -0.0009 | 0.0012 | 0.0287 | 0.0275 | -0.0163 |
| 8 | 2016/7/29 | -0.0064 | -0.0018 | -0.0435 | -0.0418 | -0.0581 |
| 9 | 2016/8/1 | -0.0139 | -0.0058 | 0.0018 | 0.0075 | -0.0505 |
| 10 | 2016/8/2 | 0.0064 | 0.0051 | 0.0256 | 0.0205 | -0.03 |
